# Fabrication of novel PVA loaded ZnO nanoparticles for anti-renal failure

**DOI:** 10.1038/s41598-024-78719-2

**Published:** 2024-11-16

**Authors:** Shawky M. Hassan, Mahmoud Tarek, Salem Samra, Shady M. El-Dafrawy

**Affiliations:** Chemistry Department, Faculty of Science, Mansoura University, Mansoura, Egypt

**Keywords:** Zinc oxide, PVA, Kidney function, Surface acidity, Nanoparticles, Coumarin synthesis, Biochemistry, Chemistry, Materials science

## Abstract

X-ray diffraction (XRD), scanning electron microscopy (SEM) and transmission electron microscopy (TEM) were used to investigate the structural, surface and particles properties of a series of zinc oxides (ZnO) doped with polyvinyl alcohol (PVA) that were prepared using the sol-gel method. The results demonstrated that the crystallinity of the catalysts decreased as the PVA content increased beyond 5 PVA/ZnO. However, on raising the calcination temperature up to 500 °C, the average crystal size of the PVA/ZnO nanoparticles increased. Next Pyridine adsorption was used to measure the surface acidity of the catalysts, and the results showed that doping ZnO with PVA and raising the calcination temperature to 500 °C increased the catalyst’s surface acidity. Furthermore, the data indicates that raising the ratio of the Brønsted to Lewis acid sites enhanced the catalytic activity for the synthesis of coumarin derivatives. We concluded that the structural and acidity characteristics of the catalysts under study had a significant impact on the catalytic activity. Furthermore, a study examining the biological activity of ZnO/PVA and pure ZnO revealed that 5PVA/ZnO performed the best in terms of improving the kidney functions of diabetic rats.

## Introduction

Environmental remediation is urgently required, particularly for wastewater. There are many strategies for achieving a clean and safe environment, among which heterogeneous photocatalysis over semiconductor nanoparticles is particularly promising^1,2^.

ZnO is one of the hardest polar inorganic materials in the family of semiconductors. It is a well-known material that has a high binding energy (60 meV) and a wide bandgap (approximately 3.2 eV). Therefore, it is considered one of the best photocatalysts for the degradation of organic pollutants. Interestingly, ZnO is non-hazardous, inexpensive, and thermally stable. In addition, it has proven to be vital in the field of catalysis and is known for its antibacterial activities^3^. Further, the nature of the active sites of ZnO is known and can be defined as Brønsted and Lewis acid sites^4^. Recently, many methods have been explored to obtain ZnO powder, including hydrothermal, gas condensation, sol–gel, and combustion methods^5,6^. The sol-gel method is a versatile chemical process used to produce solid materials from small molecules, particularly metal oxides. This technique involves the transformation of a colloidal solution (sol) into a gel-like network, ultimately leading to the formation of solid materials. Here are the key advantages of the sol-gel method compared to other fabrication techniques. Cost-Effectiveness, Low Processing Temperatures, Control Over Material Properties, Homogeneity and Purity and Environmental Benefits^7–9^.

Furthermore, ZnO has attracted considerable interest because it is a very strong acid and possesses all the advantages of heterogeneous catalysts, such as easy separation, good recovery, and reutilization. This material exhibits high catalytic activity for many important industrial reactions^10,11^. Conventionally, ZnO is synthesized by the sol–gel method^12,13^, and its properties can be improved by adding other materials to produce mixed or doped systems, which are more resistant to deactivation. Therefore, several materials, such as platinum, palladium, cetyltrimethylammonium bromide (CTAB), nickel, and polyvinyl alcohol (PVA), have been incorporated into the ZnO structure, producing catalysts with enhanced activity and selectivity^14^.

Polyvinyl alcohol (PVA) is a synthetic polymer renowned for its versatility and unique properties as, Water Solubility, Biodegradability, Chemical Resistance and Non-Toxicity which made it suitable for a wide range of applications such as, Biomedical Applications, Packaging Materials, Textile Industry, Paper Industry and Cosmetics and Personal Care Products^7,15,16^. In this work, we investigate ZnO-doped PVA nanoparticles to gain in-depth insights into the nature of the PVA/ZnO catalyst. In addition, its biological activities were investigated by studying its effect on the kidney function of diabetic rats and its catalytic performance.

## Experimental

### Materials

Oxalic acid (H_2_C_2_O_4_) (99.5%), zinc acetate dihydrate (Zn (CO_2_CH_3_)_2_ 2H_2_O) (98%), resorcinol, ethyl acetoacetate, ethanol (C_2_H_6_O) (99%), and pyridine. Were obtained from Aldrich Chemicals and used without purification. PVA (Sigma Aldrich, Mw = 86000 g/mol) was used for surface modifications. Double distilled water was used to prepare aqueous solutions.

### Preparation of catalyst

#### Preparation of ZnO nanoparticles

Sol–gel method was used to synthesize the ZnO nanoparticles. First, a solution comprising 300 mL of ethanol and 10.99 g of Zn (CO_2_CH_3_)_2_ (H_2_O)_2_ was stirred vigorously for 1 h. In another beaker, 200 mL of ethanol with 17.71 g of (H_2_C_2_O_4_) was stirred for 1 h at 50 °C and slowly added to the previous solution. Subsequently, the obtained white gelatinous precipitate was dried under a vacuum at 90 °C for 2 h, after which it was calcined at different temperatures (300 °C, 400 °C, and 500 °C).

####  Preparation of PVA/ZnO nanoparticles

To prepare 5%, 7%, and 10% PVA/ZnO nanoparticles, a solution of PVA (0.25, 0.35, and 0.5 g) in 35 mL of ethanol, respectively, was added to the Zn (CO_2_CH_3_)_2_ (H_2_O)_2_ solution (10.99 g in 300 mL of ethanol). The final precipitate was obtained using the (H_2_C_2_O_4_) solution as previously mentioned. The obtained sample was dried in a vacuum at 90 °C and calcined at the same temperatures stated above to obtain the final powder.

###  Characterization

#### X-ray diffraction (XRD)

To evaluate the formation mechanism of ZnO, the PVA/ZnO nanoparticle phases, and crystal sizes, XRD, which is a nondestructive technique, was conducted using a PW 150 (Philips) instrument, with Ni-filtered Cu Kα radiation, in the 2θ range of 10°–80º^17^. The peaks obey Bragg’s law in the estimation of the d_100_ spacing, as well as the unit cell parameter (a_o_). Bragg’s law and its relationship with the unit cell parameter are shown in Eqs. (1) and (2), respectively. The Debye–Scherrer equation (Eq. (3)) was employed in calculating the average crystalline particle size, via the XRD line-widening technique.

1 $${\text{n}}\uplambda \,=\,{\text{2}}{{\text{d}}_{{\text{1}}00}}{\text{sin }}(\uptheta ),$$

2 $${{\text{a}}_{\text{o}}}={\text{ 2}}{{\text{d}}_{{\text{1}}00}}/\sqrt 3 ,$$

3 $${\text{D}}\,=\,0.{\text{9}}\uplambda {\text{/}}\upbeta {\text{cos}}\uptheta ,$$

Where,

λ: radiation wavelength (λ = 1.54 Å); D: crystal size; β: line breadth (radiations); θ: reflection angle.

#### Microscopic studies

A Jeol-JSM-6510LV scanning electron microscope (SEM) was used to study the morphology of the catalyst surface on nano-catalysts and JEOL-JEM-2100 Transmission electron microscopy was used to measure particles size. TEM is operated at 200 kV and sample was prepared by dipping an alcoholic suspension of sample powder onto a copper grid coated with holey carbon foil then dried at ambient temperature^18^.

#### Fourier-transform infrared (FT-IR) spectroscopy of pyridine adsorption

**Scheme 1:**
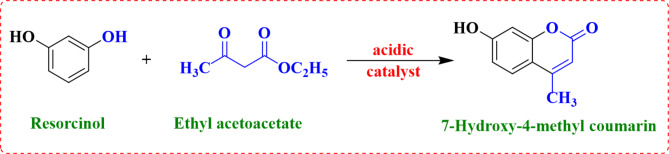
Coumarin synthesis.

The Brønsted and Lewis acid sites on the catalytic surface were examined using the FT-IR spectra of chemisorbed pyridine. Before the adsorption of pyridine^19,20^, each catalyst was activated at 120 °C for approximately 1 h. Afterward, the extra pyridine was removed by evaporation at 200 °C for 3 h under high vacuum. Thereafter, the dried pyridine was suspended for 1 month. The samples were analyzed using an FT-IR spectrophotometer, where 0.05 g of the catalyst was combined with 0.1 g of KBr in the form of a self-supporting pressed silica disk (diameter: 30 mm) (Scheme 1).

#### Catalytic activity (synthesis of 7-hydroxy-4-methyl coumarin)

A mixture of (1.1 g, 10 mmol) resorcinol and (2.5 mL, 20 mmol) ethyl acetoacetate (EAA) in a 50mL round flask was used to synthesize 7-hydroxy-4 methyl coumarin^21,22^. Subsequently, 0.1 g of the catalyst powder that was activated at 120 °C for 2 h was added to the solution.

The flask was placed in an oil bath and refluxed at 120 °C for 2 h and the reaction was monitored by TLC till completion. The resulting mixture was filtered and poured into a beaker containing crushed ice. The product was characterized by its melting point (185 °C) and FT-IR. The yield of the 7-hydroxy-4-methyl coumarin was calculated as follows:

$${\text{Yield}}\% {\text{ }}={\text{ }}({\text{Obtained Wt}}{\text{. of product}}\, \div \,{\text{Theoretical Wt}}{\text{. of product}}){\text{ }} \times {\text{ }}100$$

### Evaluation of renal failure

Before the experiment, 12 albino rats (100–120 g) were kept for adaptation under normal laboratory conditions for 7 days. All the rats were fed a balanced basal diet and randomly divided into two major groups.

Group 1 (rats with controlled diabetes): Four rats received a normal diet for 30 days without any treatment.

Group 2: Eight rats were starved for 24 h, and afterwards, they were intraperitoneally injected with streptozotocin (STZ) freshly prepared in a 0.1-M citrate buffer (pH: 4.5; dose: 4.5 mg/100 g of body weight) to induce diabetes mellitus according to a reported method^23^. Thereafter, the rats were starved for 18 h before the level of kidney function was determined.

The eight rats with diabetes of Group 2 were randomly divided into two series (four rats each), as follows.

Series 1: rats treated with ZnO, 5PVA/ZnO, 7PVA/ZnO, and 10PVA/ZnO (dose: 3.5 mg/kg of body weight).

Series 2: rats treated with ZnO, 5PVA/ZnO, 7PVA/ZnO, and 10PVA/ZnO (dose: 7 mg/kg of body weight).

Using heparinized tubes, blood samples were collected from the eye canthus of the rats every 5 days after the administration of the extracts. Each blood sample was centrifuged to obtain a clear serum, and the creatinine and the urea levels of the starved animals were determined.

- All experiments were performed in agreement with regulations of the Mansoura University, “Approved by Animal Care and Use Committee MU-ACUC, Mansoura, Egypt, and Code number: MU-ACUC (SC.MS.24.10.75)”.
- The study was conducted in compliance with the ARRIVE guidelines.

## Results and discussions

###  XRD patterns of PVA/ZnO

**Fig. 1:**
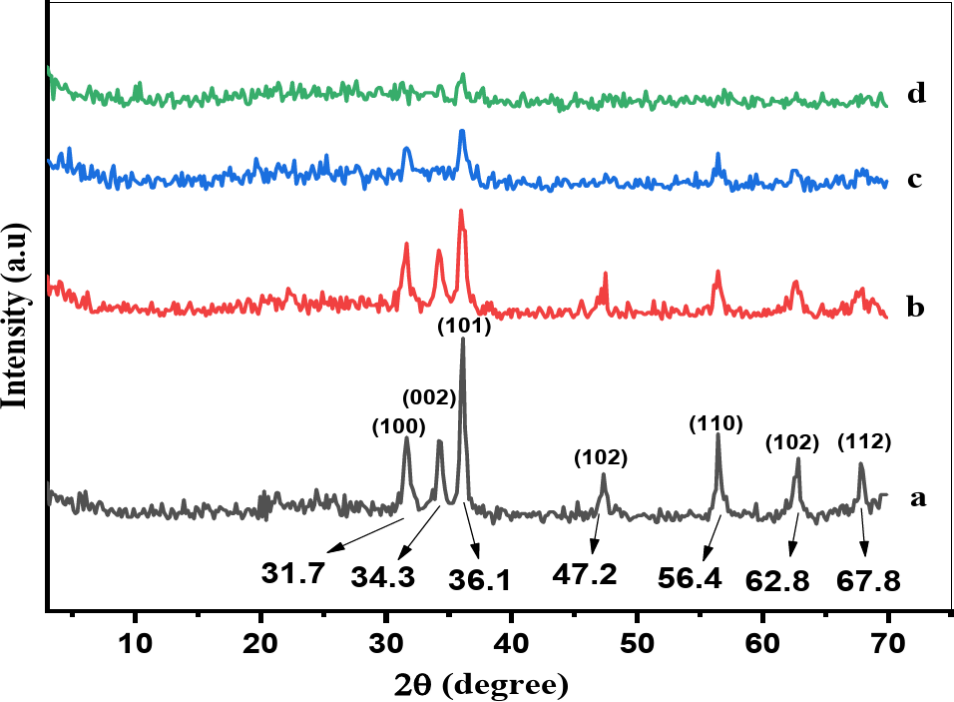
XRD pattern of (**a**) ZnO, (**b**) 5PVA/ZnO, (**c**) 7PVA/ZnO and (**d**) 10PVA/ZnO catalysts calcined at 400 °C.

**Fig. 2:**
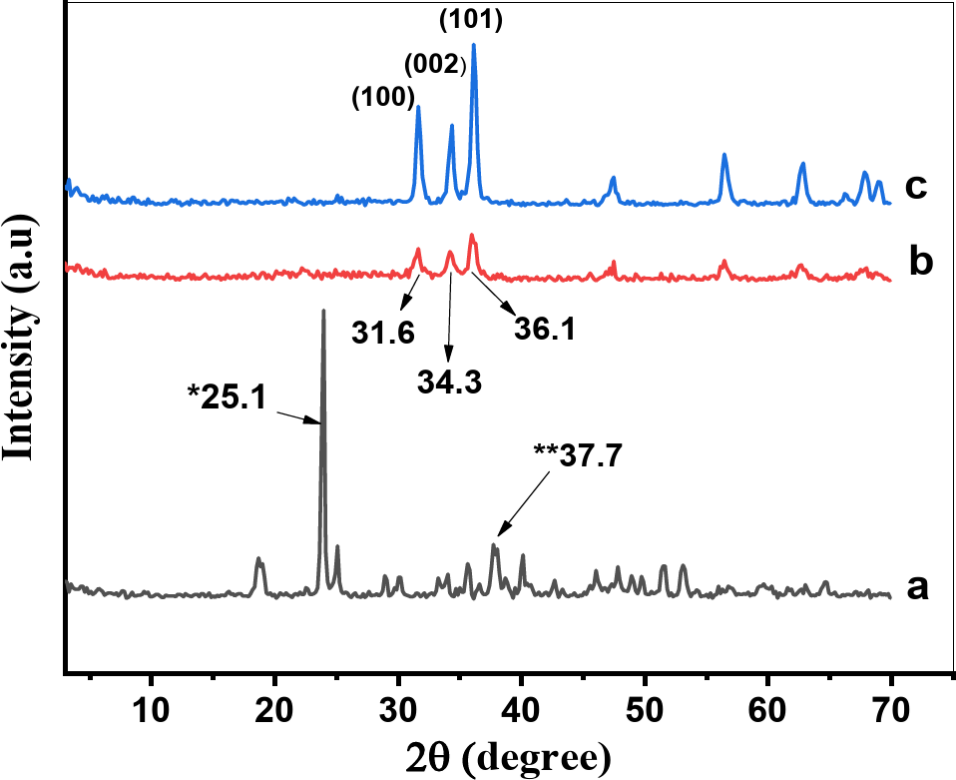
XRD pattern of ZnO catalysts at (**a**) 300, (**b**) 400 and (**c**) 500 °C.

**Fig. 3:**
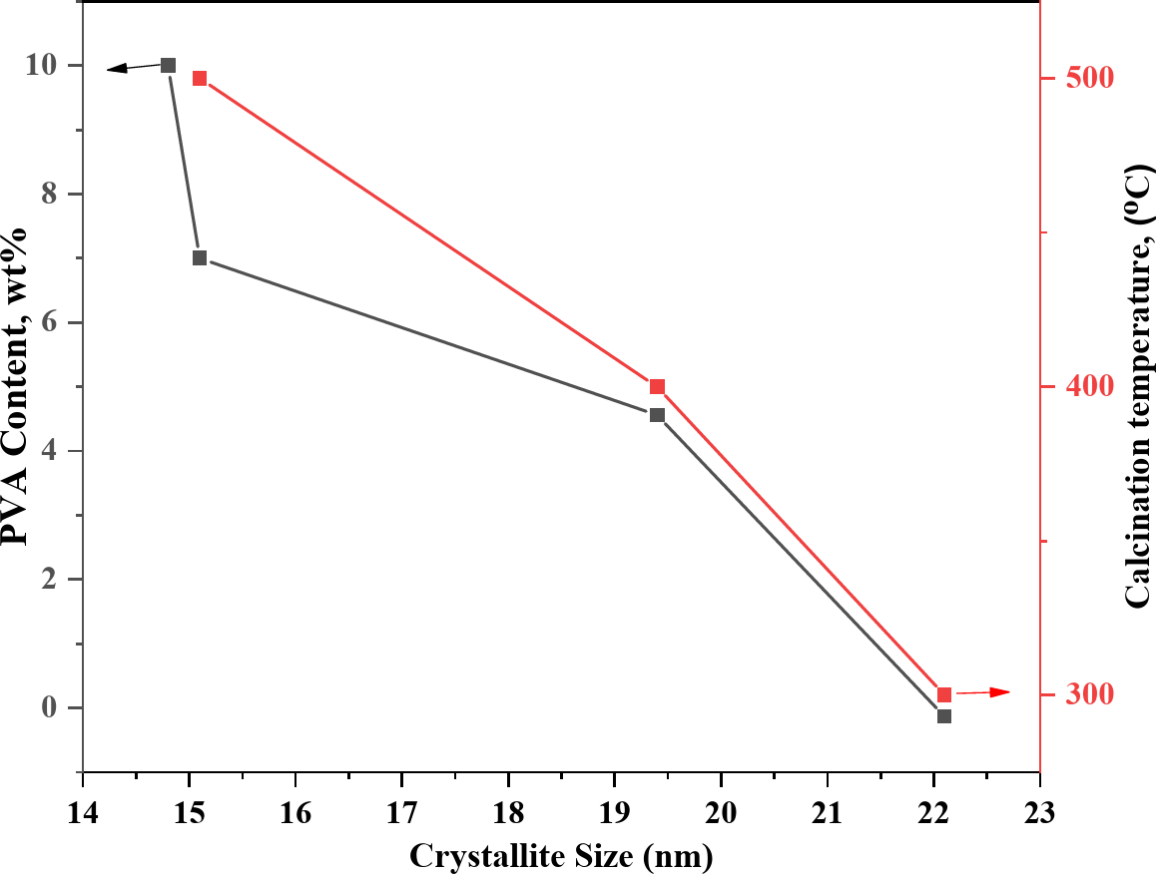
Effect of PVA content in ZnO samples calcined at 400 °C and calcination temperatures in 5PVA/ZnO samples on crystallite size (of peak 2θ = 36.1°).

By using the XRD patterns Figs. 1 and 2; Table 1 and comparing with JCPDS card number (043 − 0002) of zinc oxide^24^, it is showing that the effects of the PVA concentration and calcination temperatures on the crystal size of the synthesized samples were determined Fig. 3. We observed that all the samples possessed a typical hexagonal structure^25^, no phase was observed for PVA. As shown in Fig. 1, the intensities of the (101), (002), and (100) peaks at 2θ = 36.1°, 34.3°, and 31.6°, respectively, decreased as the PVA content increased because of the increase in the degree of crystal deformation.

As shown in Fig. 2, at 300 °C, the peaks of Zn (CO_2_CH_3_)_2_ (H_2_O)_2_ with a monoclinic structure were formed at 2θ = 25.1°, while the peaks of Zn (CO_2_CH_3_)_2_ with a monoclinic structure were formed at 2θ = 37.7°. By increasing the temperature of 5PVA/ZnO to 400 °C and 500 °C, the intensities of the peaks at 2θ = 36.1°, 34.3°, and 31.6°, were increased respectively, this is because the PVA content decreased when the temperature increased, and the ZnO phase was maintained. The average crystal size of PVA/ZnO nanoparticles at 300 °C, 400 °C, and 500 °C increased and was estimated using the Scherrer equation^26^ (Eq. (1)):

$${{\text{D}}_{{\text{1}}0{\text{1}}}}=K\lambda /\beta \cos \theta$$

Where,

D_101_: crystallite size; K: constant usually taken as 0.94; λ: radiation wavelength of the X-ray; β: line width at half maximum height; θ: Bragg angle.

The crystallite sizes of the hexagonal phase line at 2θ = 36.1° is reflexed for the selected samples is summarized in Table 1, which shows the effect of the PVA content on the crystallite size of ZnO and calcination temperature on 5PVA/ZnO. Figure 3 shows that, the crystallite size of the hexagonal phase decreased from 22.1 to 14.8 nm as the PVA content increased. While by increasing calcination temperature of 5PVA/ZnO at 2θ = 36.1° from 300 °C to 500 °C, the crystallite size increased from 17.6 to 29.3 nm. This is attributed to the evaporation of some PVA upon increasing the thermal energy. In all cases, the average crystal size of the treated PVA/ZnO was less than that of pure one.

**Table 1:** Average crystallite size of different PVA concentration samples and different calcination temperatures at 2θ = 36.1°.

Peak	Sample name/calcination	Average crystallite size (nm)
36.1	ZnO/300 °C	–
	ZnO/400 °C	22.1
	ZnO/500 °C	–
36.1	5PVA/ZnO/300 °C	17.6
	5PVA/ZnO/400 °C	19.4
	5PVA/ZnO/500 °C	29.3
36.1	7PVA/ZnO/300 °C	–
	7PVA/ZnO/400 °C	15.1
	7PVA/ZnO/500 °C	–
36.1	10PVA/ZnO/300 °C	–
	10PVA/ZnO/400 °C	14.8
	10PVA/ZnO/500 °C	–

### Microscopic studies

#### SEM analysis

**Fig. 4:**
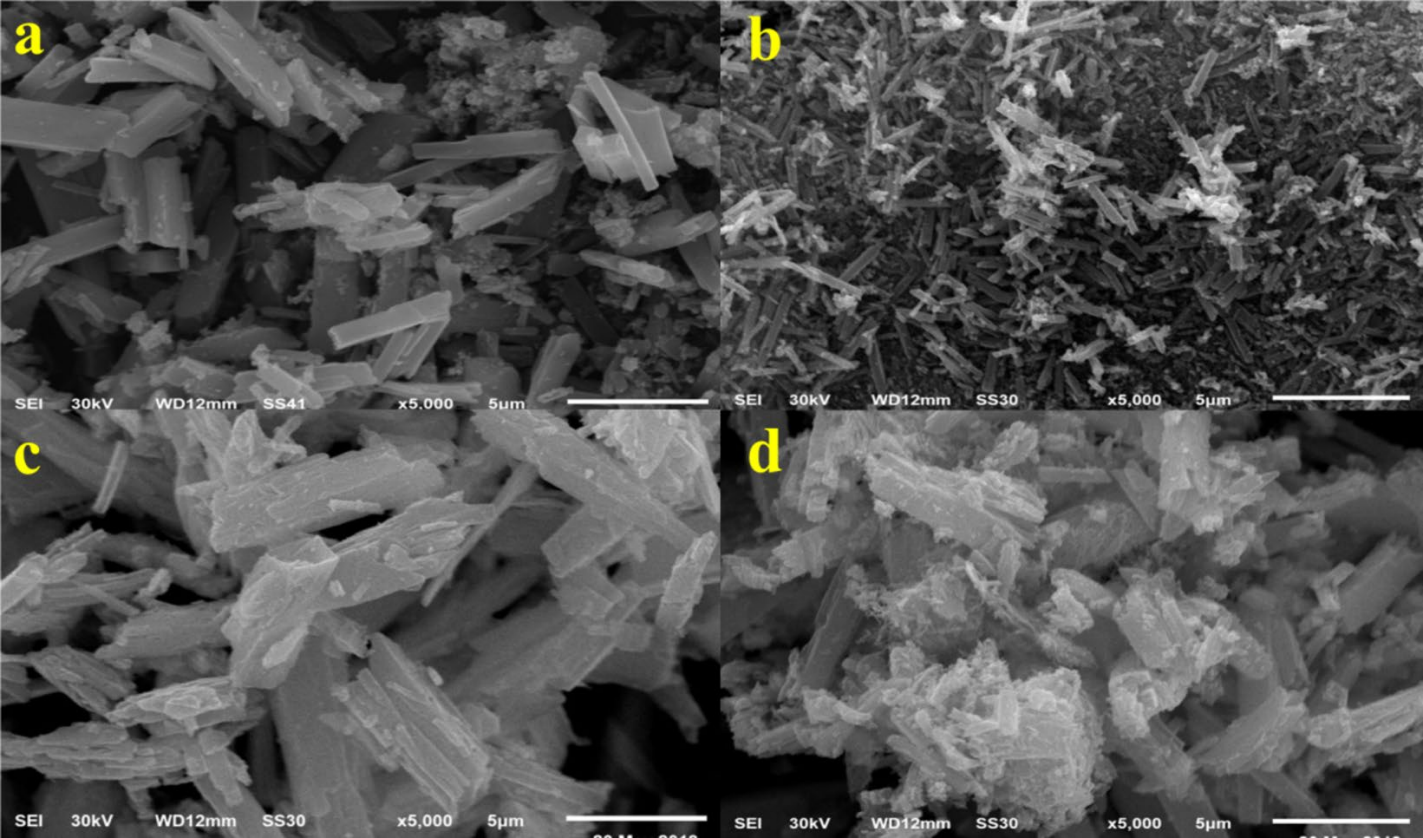
SEM images of (**a**) ZnO, (**b**) 5PVA/ZnO, (**c**) 7PVA/ZnO and (**d**) 10PVA/ZnO calcined at 400 °C.

Figure 4 shows SEM images of ZnO and ZnO/PVA catalysts with various content of PVA 5, 7 and 10% which reveals that increasing PVA content, increases hexagonal phase and the small crystals with various phases were interwoven with each other creating strongly bound nanoclusters^27,28^.

#### TEM analysis

**Fig. 5:**
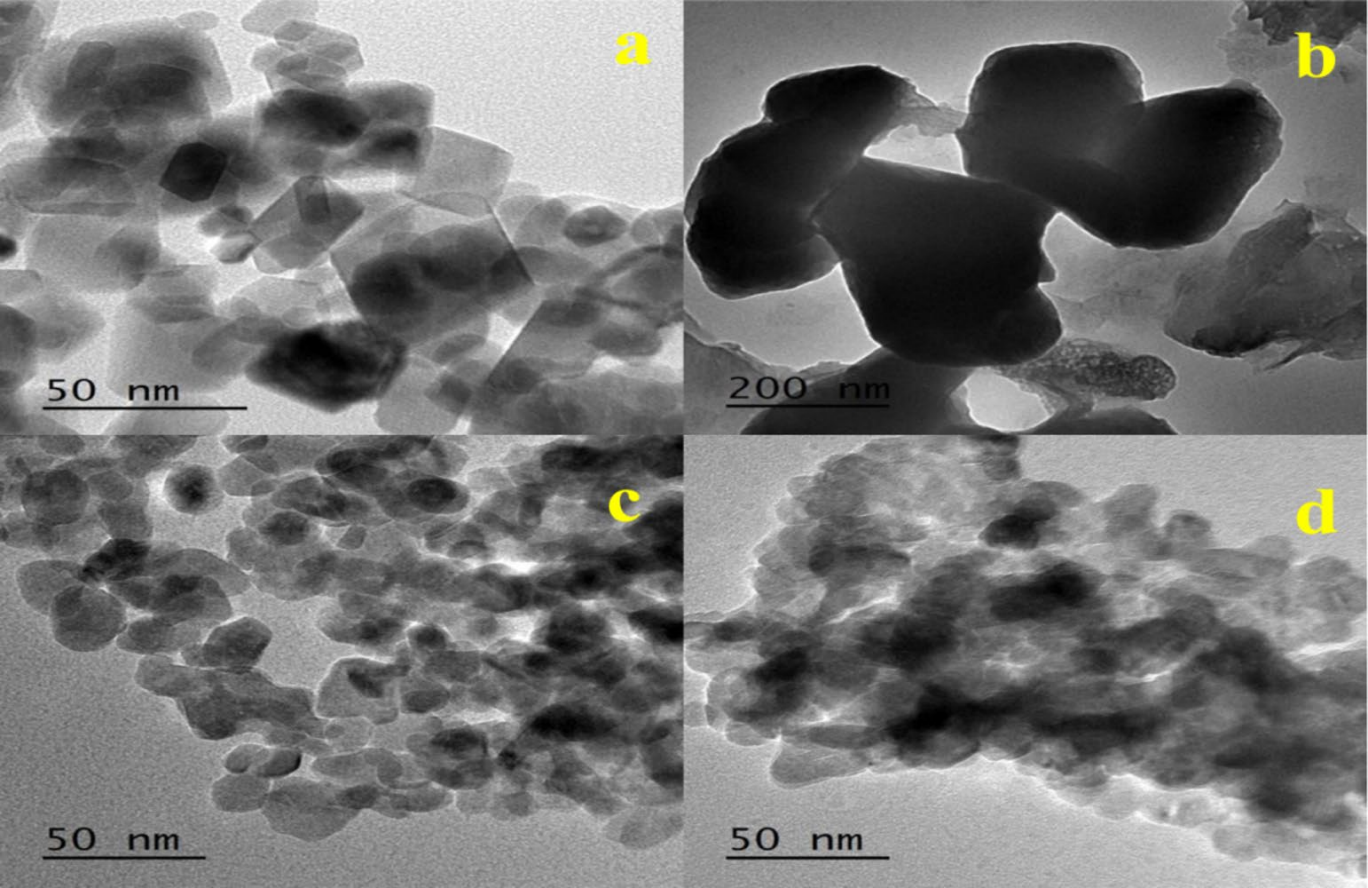
TEM images of (**a**) ZnO, (**b**) 5PVA/ZnO, (**c**) 7PVA/ZnO and (**d**) 10PVA/ZnO calcined at 400 °C.

TEM images Fig. 5 shows that there are different particles size range of hexagonal nanoparticles architecture. This wide range of ultra-small particles size introduce some important benefits as high catalytic activity and surface area.

### Surface acidity of PVA/ZnO by pyridine adsorption

#### FT-IR

Pyridine adsorption is an effective method for investigating Lewis and Brønsted acid sites occurred at different wavenumbers because of the difference in the bonding nature. The percentage of the Brønsted and Lewis acid sites was calculated from following relationships^29–31^:

$$\% {\text{Bronsted }}=\left( {{{\text{A}}_{\text{B}}}/{{\text{A}}_{\text{L}}}+{\text{ }}{{\text{A}}_{\text{B}}}} \right){\text{ }} \times {\text{ }}100{\text{ and }}\% {\text{Lewis }}=\left( {{{\text{A}}_{\text{L}}}/{{\text{A}}_{\text{L}}}+{\text{ }}{{\text{A}}_{\text{B}}}} \right){\text{ }} \times {\text{ }}100$$

Where **A**_**B**_ is the integrated area of the Brønsted acid site peaks, and **A**_**L**_ is that of the Lewis acid site peaks.

**Fig. 6:**
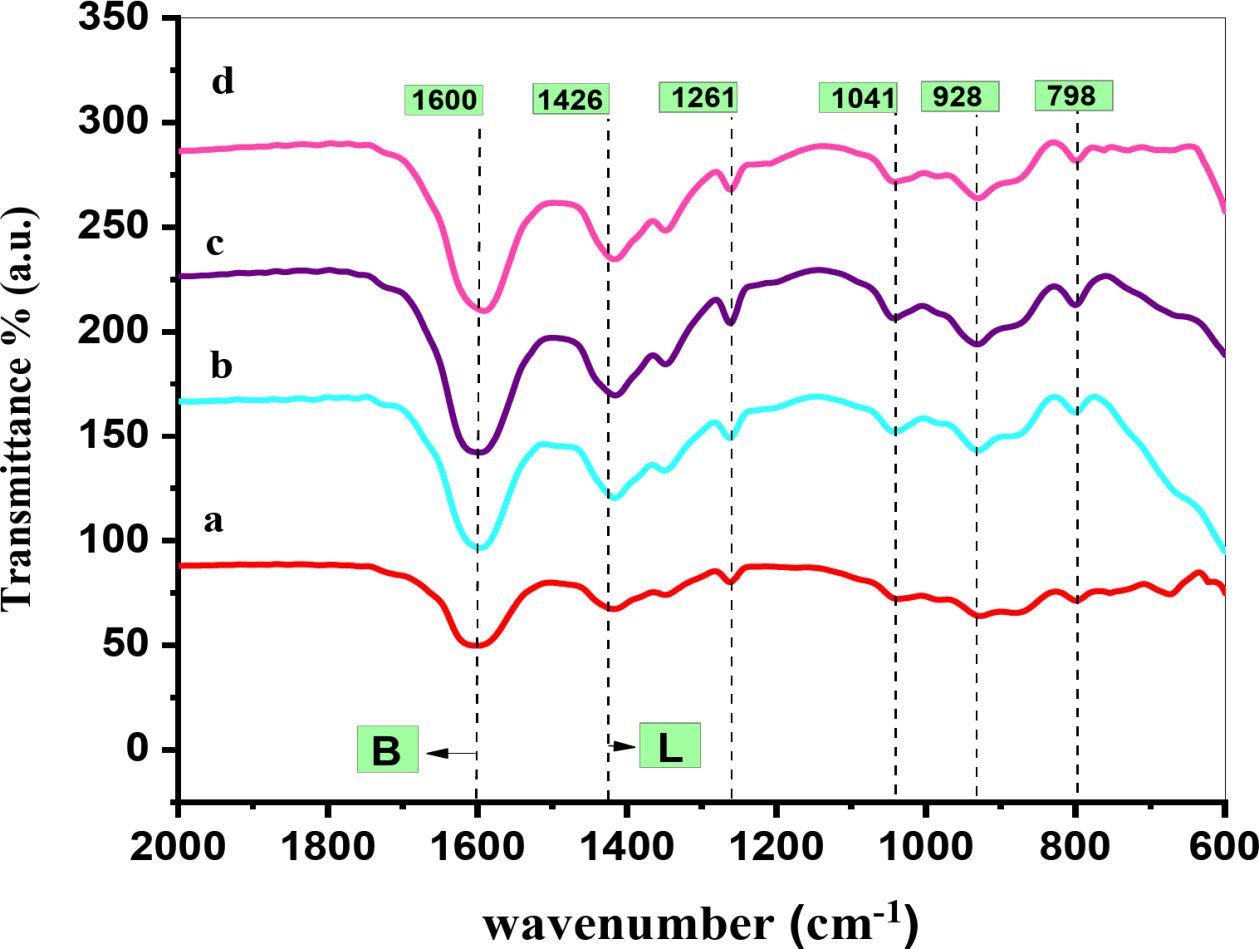
FT-IR spectra of chemisorbed pyridine on (**a**) ZnO, (**b**) 5PVA/ZnO, (**c**) 7PVA/ZnO and (**d**) 10PVA/ZnO at calcination 500 °C.

**Fig. 7:**
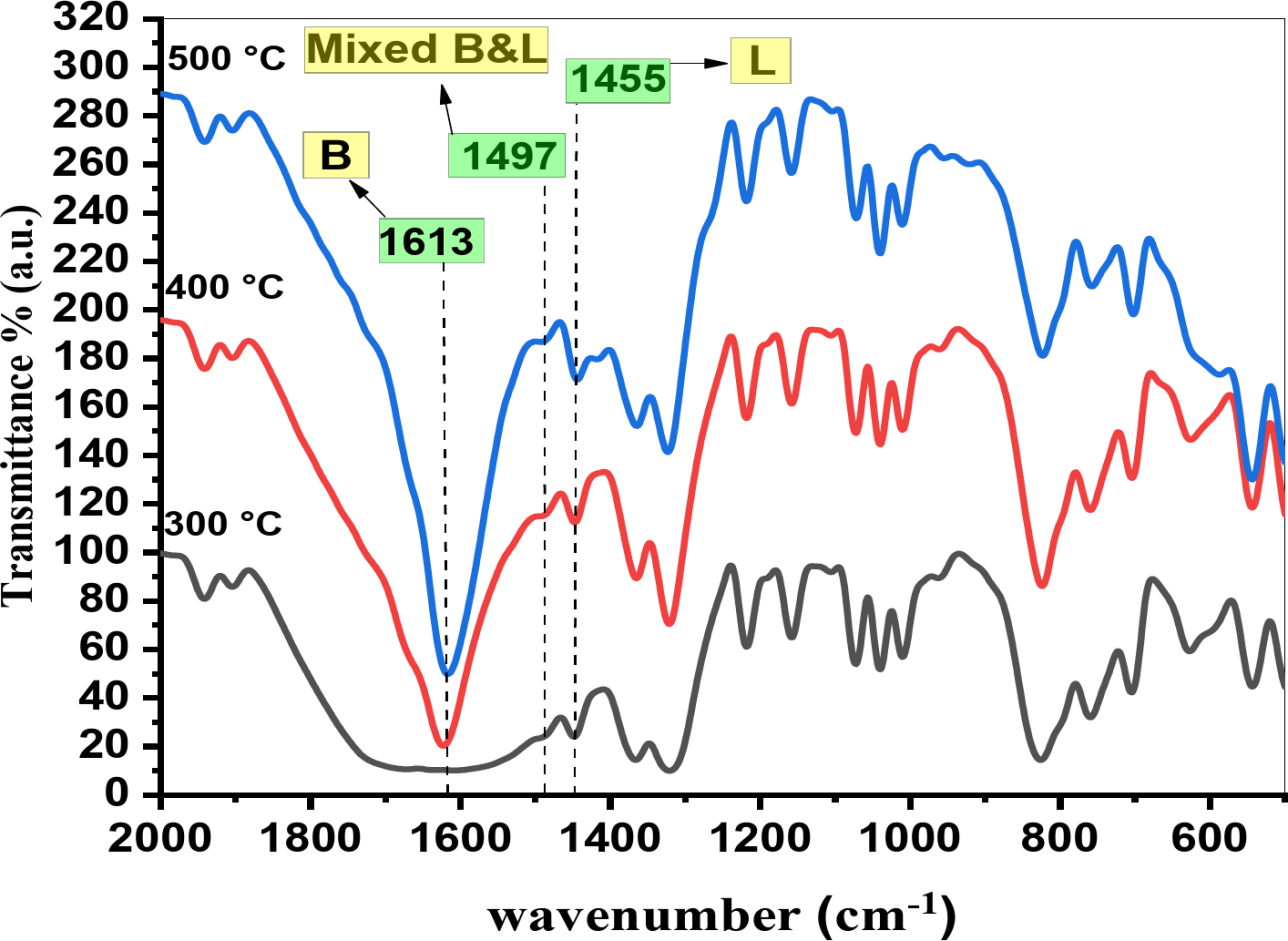
FT-IR spectra of chemisorbed pyridine on 5PVA/ZnO at different calcination.

All the catalysts investigated showed the bands of the adsorbed pyridine around 1426 cm^− 1^, assigned to the pyridine coordinated to Lewis acid sites while at 1600 cm^− 1^ and 1626 cm^− 1^, assigned to the adsorbed pyridinium ions (pyH^+^) on Brønsted acid sites. However, the interaction of the pyridine species with mixed Lewis and Brønsted acid sites produced a band at around 1497 cm^− 1^, Fig. 6^32–34^. Also, there are four bands appears near 798, 928, 1041 and 1261 cm^− 1^ which may be corresponding to Zn-O bond, C-H aliphatic, C = C bond and O-H bond. By increasing the concentration of PVA up to 10%, the intensities of these peaks increased. While Fig. 7 shows the FT-IR spectra of the chemisorbed pyridine on 5PVA/ZnO at different calcination temperatures and reveals that by increasing the calcination temperature, the intensities of the Brønsted and Lewis acid sites peaks increased. From the beginning of the calcination at 400 °C, a new peak appeared at 1497 cm^− 1^, which was assigned to the mixed Lewis and Brønsted acid sites.

**Fig. 8:**
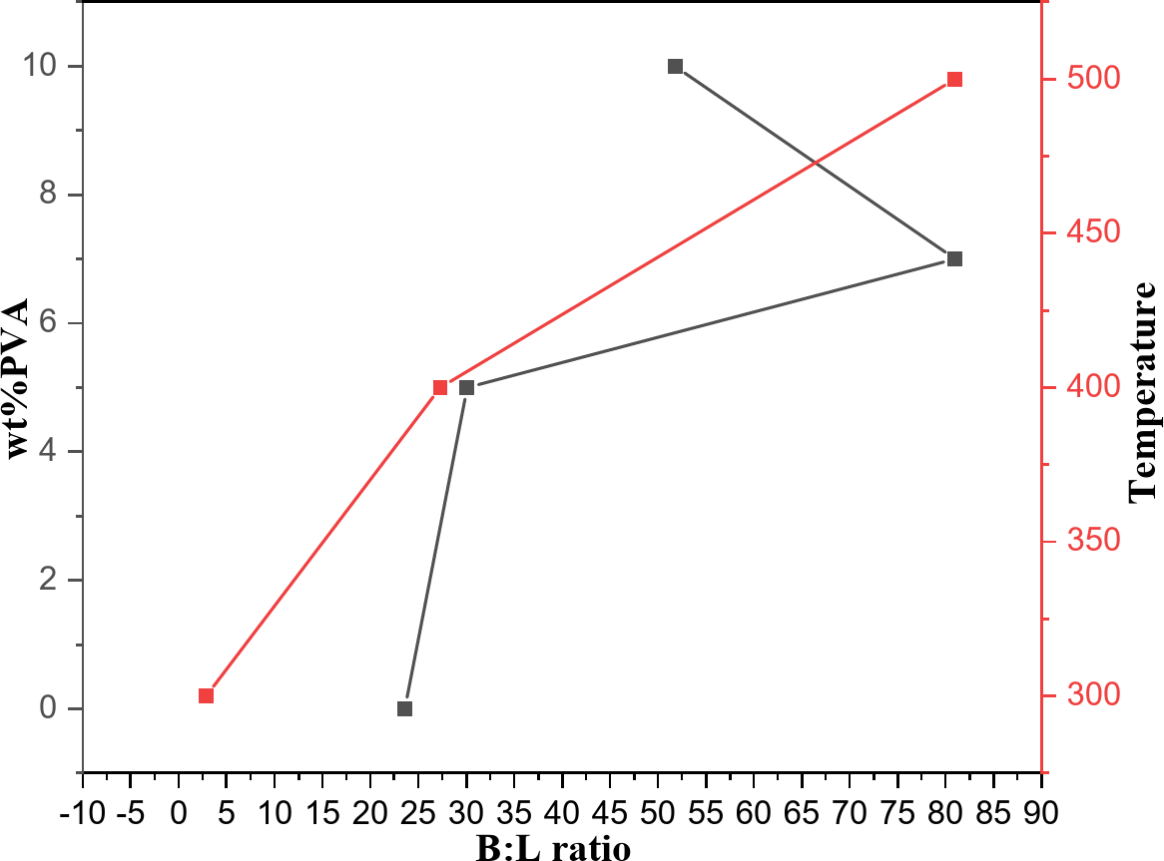
Effect of PVA content at calcination 500 °C and effect of calcination temperatures over 5PVA/ZnO catalyst on Brønsted to Lewis acid sites ratio (B/L ratio).

Figure 8 explains the relationship between B/L ratio and the PVA content. It was revealed that the 5PVA/ZnO calcined at 500 °C possessed an optimum B/L ratio. A further increase in the PVA content decreased the B/L ratio. This can be attributed to the aggregation of PVA, which reduced the availability of the Brønsted and Lewis acid sites to pyridine. Karim et al. achieved similar results for WO_3_ loaded on ZrO_2_^35^. And also, at the same figure there are relationship between the Brønsted to Lewis acid sites ratio (B/L ratio) and the calcination temperature over the 5PVA/ZnO catalyst. It is observed that the B/L ratio increases with the calcination temperature up to 500 °C. This may be attributed to the aggregation of PVA at relatively high temperatures, which lowers the accessibility of the Lewis and Brønsted acid sites to pyridine.

### Synthesis of 7-hydroxy-4-methyl coumarin

Pechmann condensation of resorcinol and ethyl acetoacetate was conducted to yield coumarin derivative^36–38^, using pure ZnO and doped ZnO with different concentrations of PVA.

#### Effect of PVA concentration

**Fig. 9:**
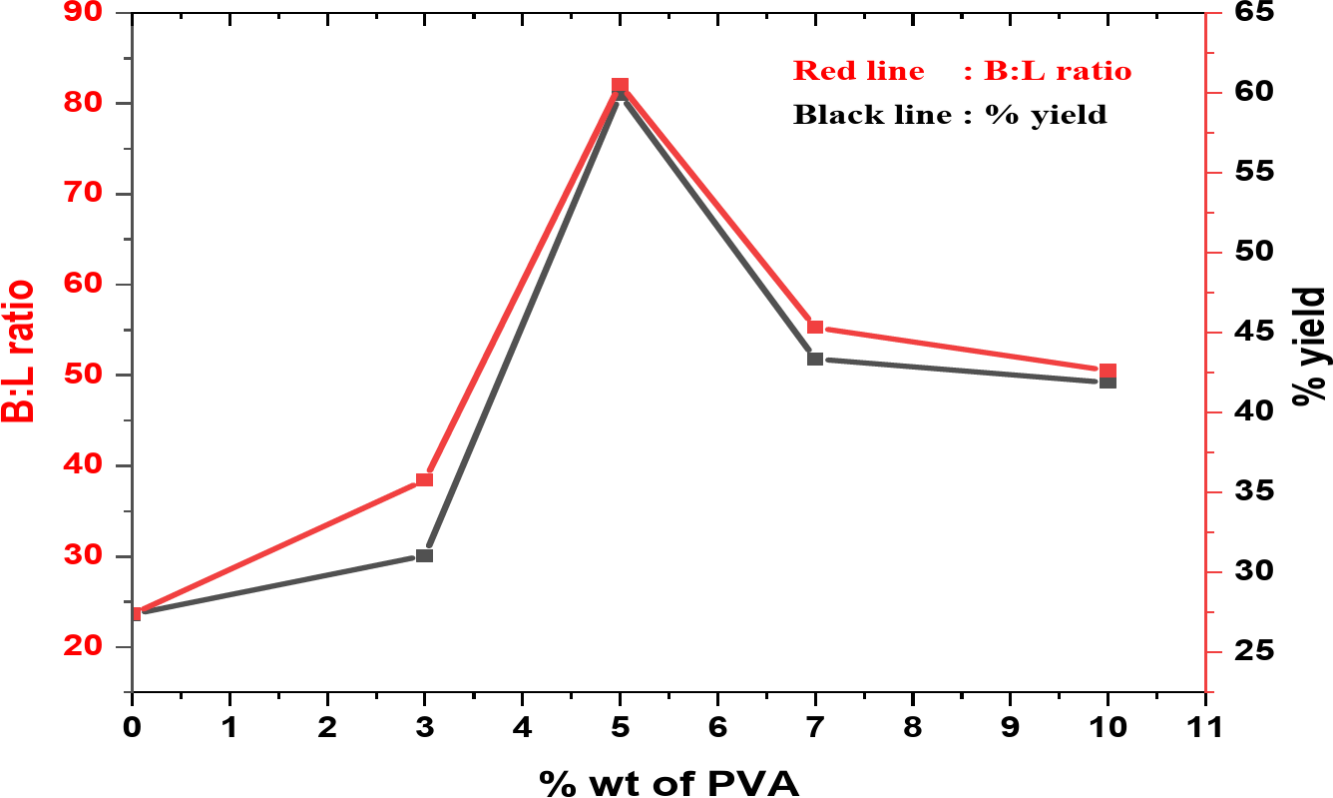
Effect of PVA content (wt %) on (B/L) ratio and yield % of 7-hydroxy-4-methyl coumarin at calcination 500 °C.

Figure 9 shows that 5PVA/ZnO catalyst is the most active catalyst among the investigated catalysts and this result was confirmed by XRD and pyridine adsorption results, which indicated that 5PVA/ZnO possessed the largest crystallite size and maximum B/L ratio.

####  Effect of molar ratio of reactants using 5PVA/ZnO

**Fig. 10:**
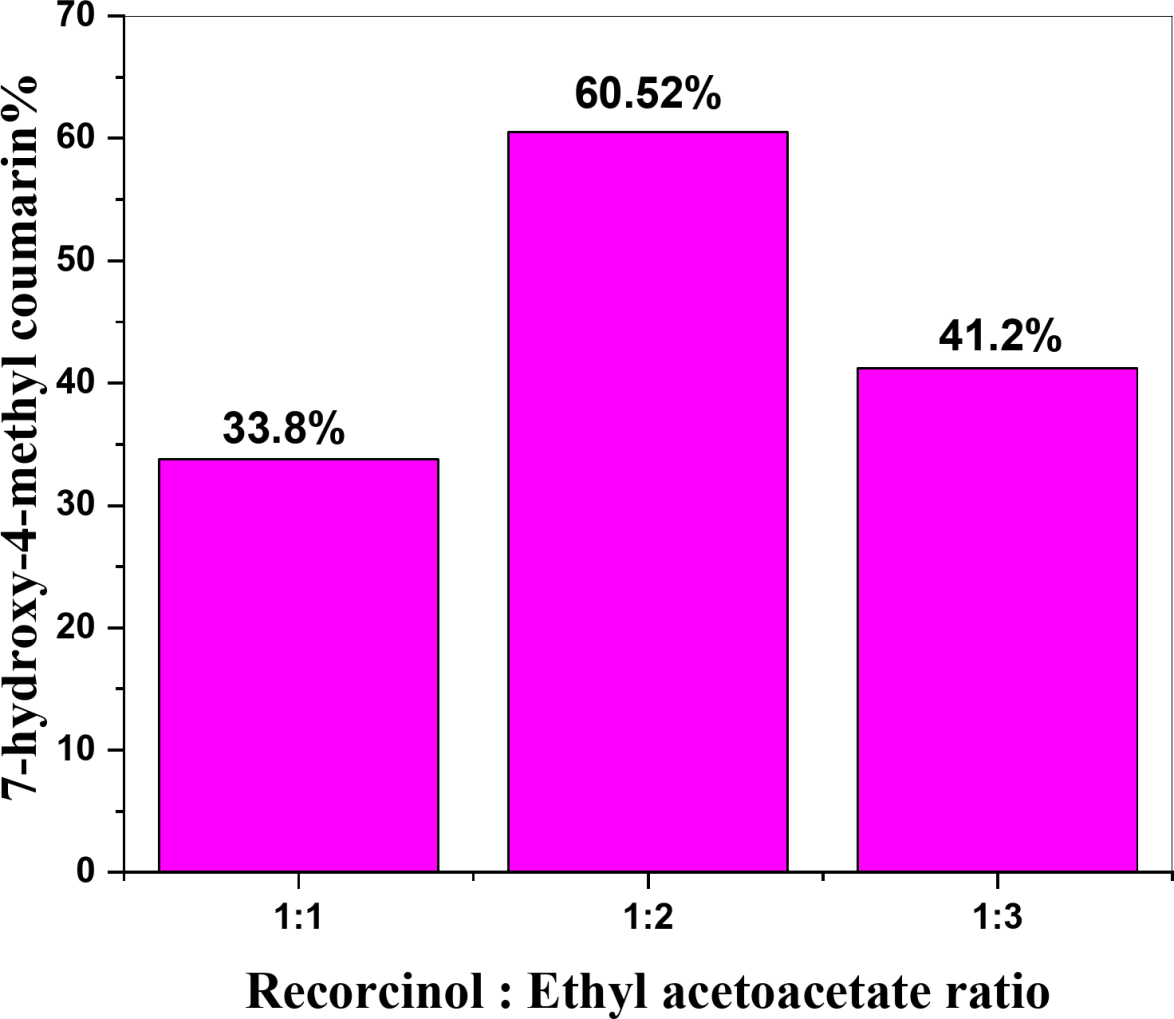
Effect of molar ratio of reactants on yield of % 7-hydroxy-4-methyl coumarin over 5PVA/ZnO catalyst calcined at 500 °C.

Figure 10 shows that the coumarin yield increased from 33.8 to 60.52% when the molar ratio of resorcinol: ethyl acetoacetate increased from 1:1 to 1:2. Afterward, it decreased to 41.2% when the ratio was increased to 1:3.

This decrease may be attributed to the saturation of the catalyst surface with ethyl acetoacetate, which blocked the acid sites, thereby reducing the catalyst efficiency^39,40^.

####  Effect of calcination temperatures

Evidently, the synthesis of 7-hydroxy-4-methyl coumarin strongly depends on the calcination temperature, which can affect the crystalline structure as well as acidic and surface characteristics.

**Fig. 11:**
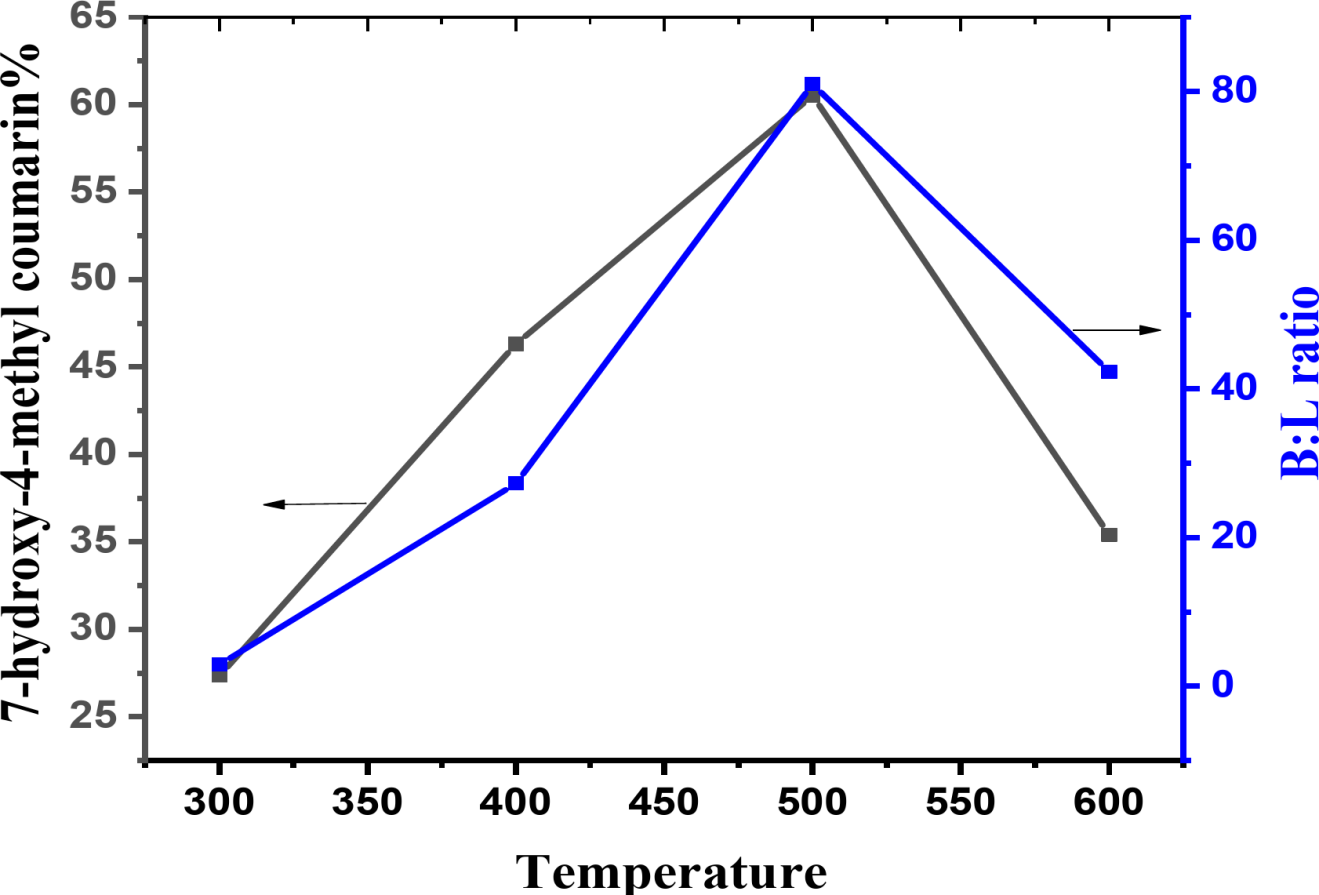
Effect of calcination temperature on (B/L) ratio and yield % of 7-hydroxy-4-methyl coumarin over 5PVA/ZnO.

Figure 11, shows that the highest yield was achieved at calcination temperature 500 °C. This can be attributed to the B/L ratio, which increased as the calcination temperature increased.

### Evaluating renal failure of diabetic rats

**Fig. 12:**
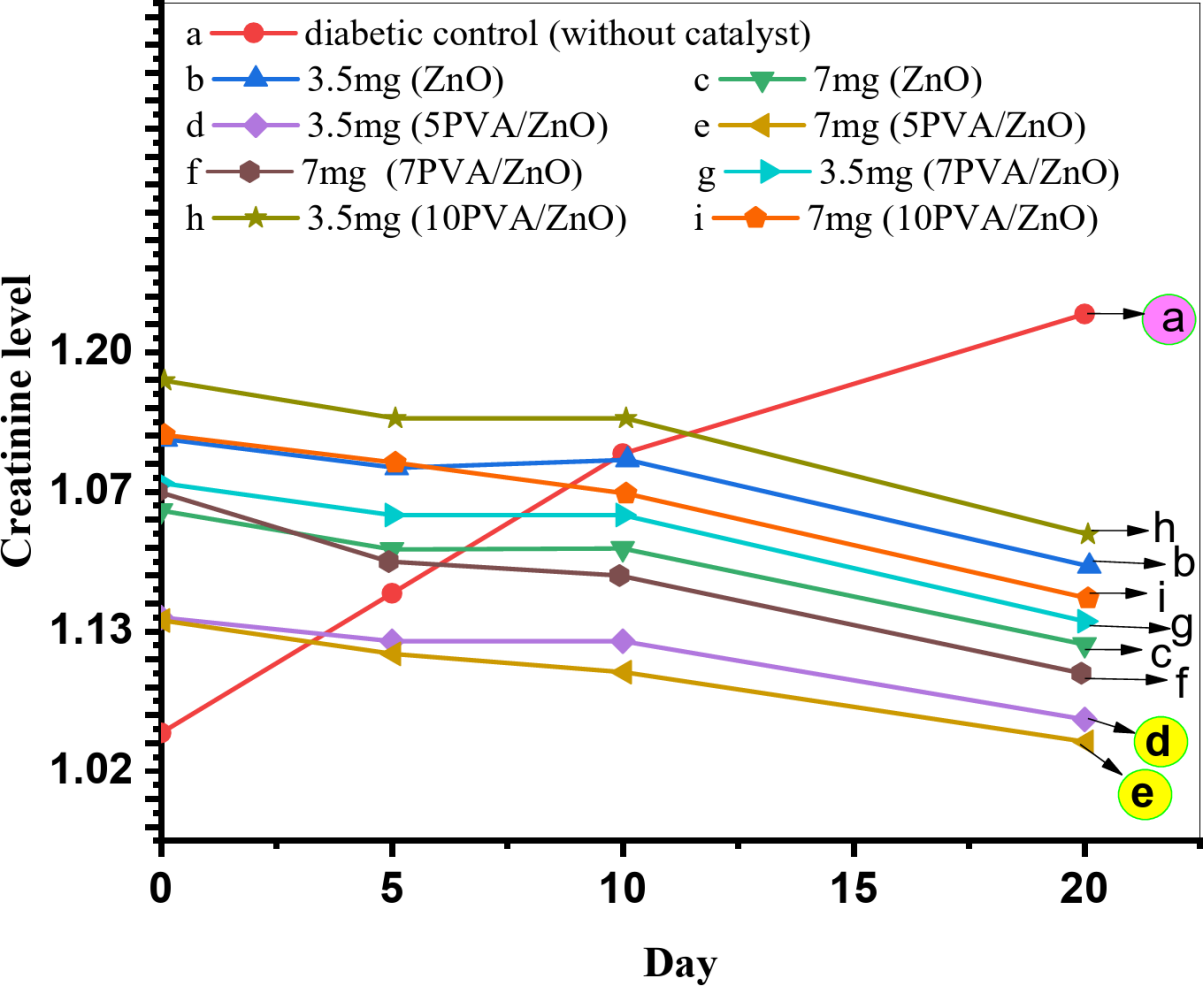
Relation between creatinine level and number of days for different% of PVA calcined at 300 °C for two series 3.5 mg and 7.0 mg.

**Fig. 13:**
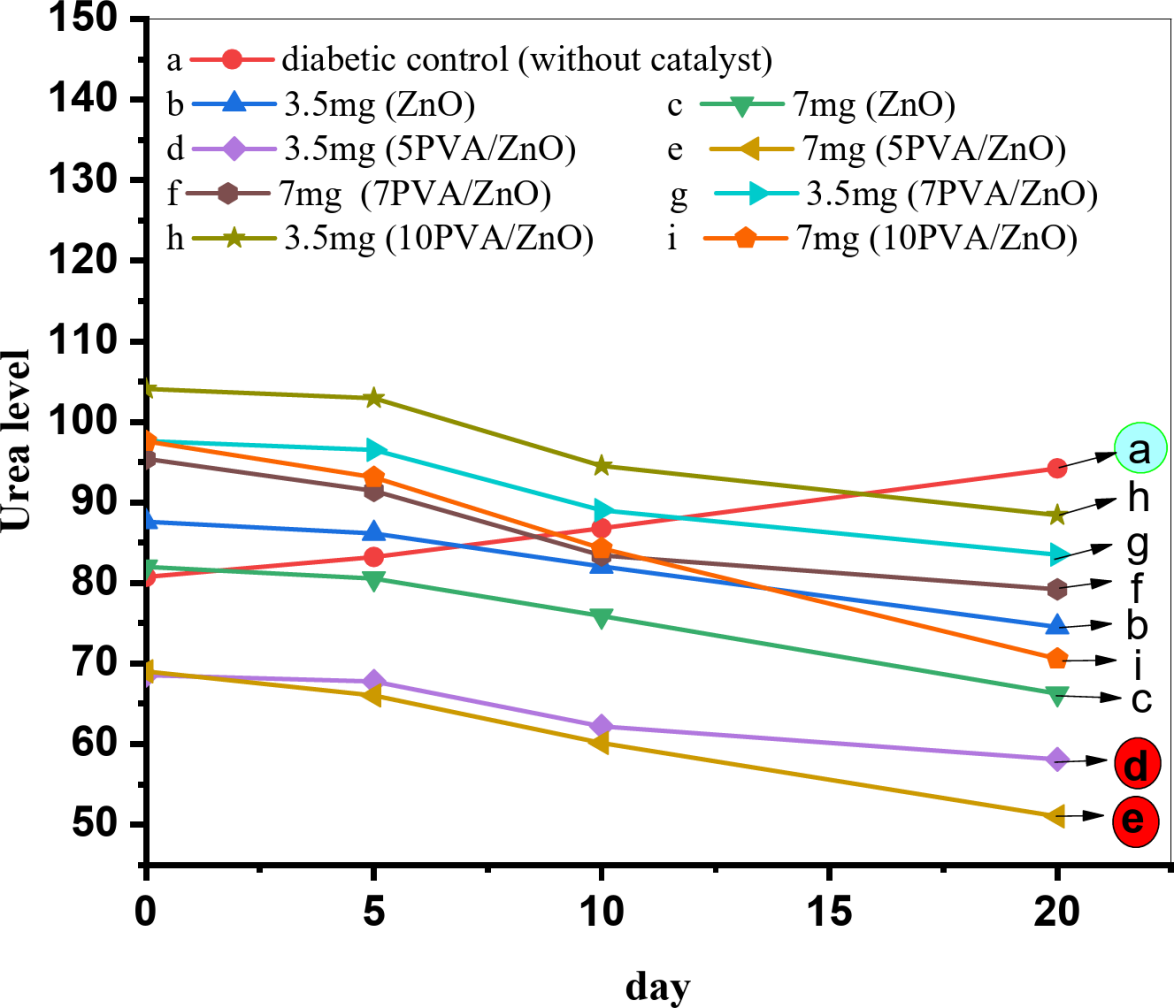
Relation between Urea level and number of days for different% of PVA calcined at 300 °C for two series 3.5 mg and 7.0 mg.

Figures 12 and 13; Tables 2 and 3 shows the effect and results of the catalysts on creatinine and urea levels for two series of diabetic rats 3.5 mg and 7.0 mg of catalyst, as it obvious from the figs and tables, by comparing the kidney functions in diabetic control rats without catalyst by two series of rats which injected by the catalysts, we were noticed that there are a dramatically improvement in kidney functions especially for rats injected by 5PVA/ZnO in two series, it were decreased to 1.53 and 1.42 in creatinine level where as 57.99 and 51.01 in urea level^41^.

**Table 2:** Creatinine level at different days and% of PVA for two series 3.5 mg and 7.0 mg.

Creatinine level	Treatment period	Normal control	Diabetic control	ZnO dose	
				3.5 mg	7 mg
at ZnO	Zero time	1.02 ± 03	2.24 ± 01	3.1132	2.933
	5 days	1.13 ± 00	2.94 ± 13	2.908	2.654
	10 days	1.07 ± 05	3.09 ± 01	2.9666	2.660
	20 days	1.20 ± 00	3.27 ± 07	2.2028	1.973
at 5PVA/ZnO	Zero time	1.02 ± 03	2.24 ± 01	2.26	2.29
	5 days	1.13 ± 00	2.94 ± 13	2.09	2.05
	10 days	1.07 ± 05	3.09 ± 01	2.09	1.92
	20 days	1.20 ± 00	3.27 ± 07	**1.53**	**1.42**
at 7PVA/ZnO	Zero time	1.02 ± 03	2.24 ± 01	3.05	3.2
	5 days	1.13 ± 00	2.94 ± 13	2.82	2.7
	10 days	1.07 ± 05	3.09 ± 01	2.82	2.6
	20 days	1.20 ± 00	3.27 ± 07	2.06	1.9
at 10PVA/ZnO	Zero time	1.02 ± 03	2.24 ± 01	3.4	3.3
	5 days	1.13 ± 00	2.94 ± 13	3.13	3.1
	10 days	1.07 ± 05	3.09 ± 01	3.13	2.88
	20 days	1.20 ± 00	3.27 ± 07	2.3	2.13

**Table 3:** Urea level at different days and% of PVA for two series 3.5 mg and 7.0 mg.

Urea level	Treatment period	Normal control	Diabetic control	ZnO dose	
				3.5 mg	7 mg
at ZnO	Zero time	47.13 ± 14	68.97 ± 01	90.374	85.84
	5 days	48.99 ± 11	71.42 ± 05	88.942	84.40
	10 days	48.00 ± 01	75.00 ± 01	84.843	79.74
	20 days	47.80 ± 00	82.44 ± 09	77.330	70.09
at 5PVA/ZnO	Zero time	47.13 ± 14	68.97 ± 01	68.44	69.00
	5 days	48.99 ± 11	71.42 ± 05	67.66	66.02
	10 days	48.00 ± 01	75.00 ± 01	62.08	60.14
	20 days	47.80 ± 00	82.44 ± 09	**57.99**	**51.01**
at 7PVA/ZnO	Zero time	47.13 ± 14	68.97 ± 01	92.4	93.1
	5 days	48.99 ± 11	71.42 ± 05	91.3	89.1
	10 days	48.00 ± 01	75.00 ± 01	83.8	81.1
	20 days	47.80 ± 00	82.44 ± 09	78.3	76.9
at 10PVA/ZnO	Zero time	47.13 ± 14	68.97 ± 01	102.66	103.5
	5 days	48.99 ± 11	71.42 ± 05	101.49	99.03
	10 days	48.00 ± 01	75.00 ± 01	93.1	90.21
	20 days	47.80 ± 00	82.44 ± 09	86.98	76.5

## Conclusions

ZnO and PVA/ZnO nanoparticles were synthesized using the Sol-gel method, and their properties were assessed by XRD analysis, which demonstrated that the catalysts had crystallized in a hexagonal structure. SEM and TEM analysis were used to describe the surface morphology and particles size, while pyridine adsorption was used to detect surface acidity. The catalytic activity of the catalysts was assessed by synthesizing coumarin derivatives, and its biological activity was ultimately inferred by assessing their impact on diabetic rats’ renal failure. The analysis mentioned indicates that the 5PVA/ZnO catalyst that was calcined at 300 °C showed the lowest levels of creatinine at 1.53 and 1.42 while urea level became 57.99 and 51.01 among the members of the two series. On the other hand, 5PVA/ZnO catalyst that was calcined at 500 °C demonstrated the highest crystallite size, B/L ratio for surface acidity in pyridine adsorption, and the best coumarin yield.

## Data Availability

All data needed to support the conclusions are present in the paper. Additional data related to this paper may be requested from shady M. El Dafrawy .
